# Outcomes of Pressure Ulcer Injuries Classified by Race: A 10-Year Nationwide Analysis

**DOI:** 10.7759/cureus.71097

**Published:** 2024-10-08

**Authors:** Andrej M Sodoma, Spencer Shain, Muhammad Wahdan Naseeb, Samuel Greenberg, Argirios Skulikidis, Sadia Arshad

**Affiliations:** 1 Internal Medicine, South Shore University Hospital, Bay Shore, USA; 2 Urology, New York Institute of Technology College of Osteopathic Medicine (NYITCOM), Old Westbury, USA; 3 Anesthesia and Critical Care, Stony Brook University, Stony Brook, USA

**Keywords:** distributive shock, heel wounds, inclusion and diversity, pressure wounds, preventative care, sacral ulcer, severe sepsis, skin injury

## Abstract

Objectives: Pressure ulcer injuries (PIs) are ischemic changes to the skin caused by long-term pressure on bony prominences. This study aimed to investigate the prevalence of PIs and their effects on minority groups in the hospital setting in the United States.

Methods: The National Inpatient Sample (NIS) from 2011 to 2020 was used to identify adults hospitalized in the United States who received a diagnosis of PI. International Classification of Diseases, Ninth Revision (ICD-9) and Tenth Revision (ICD-10) codes were used to select patients. An equal number of random records, stratified by year and without a diagnosis of PI, were selected to serve as controls. Records were analyzed for baseline characteristics using a chi-square test.

Results: Adjusted odds ratios (ORs) of developing a pressure ulcer were calculated using multivariate logistic regression, a weighted total of 5,993,667 PI admissions were included in this study, and 5,993,667 non-PI admissions were included. PI patients were more likely than controls to be male (OR = 0.88; 95% confidence interval {CI}: 0.88, 0.89; p < 0.05). PI patients were more likely to be older than non-PI patients (75+ years; OR = 3.46; 95% CI: 3.38, 3.54; p < 0.05). PI patients were more likely to be on Medicare or Medicaid (OR = 1.94; 95% CI: 1.92, 1.97; p < 0.05) than private insurance. PI patients were far more likely to have a high Charlson Comorbidity Index (CCI) of 3+ (OR = 10.44; 95% CI: 10.18, 10.71; p < 0.05) than a lower CCI score. Compared to Whites, African Americans (OR = 1.49; 95% CI: 1.47, 1.51; p < 0.05) were at higher risk of PIs. Among PI patients, White patients had a lower risk of death compared to African Americans (OR = 1.09; 95% CI: 1.07, 1.11; p < 0.05). African Americans had lower rates of acute kidney injury (AKI) compared to Whites (OR = 0.88; 95% CI: 0.86, 0.91; p < 0.05). Compared to Whites, rates of sepsis were higher for African Americans (OR = 1.40; 95% CI: 1.38, 1.42; p < 0.05).

Conclusion: A racial discrepancy in pressure ulcer prevalence was shown in racial minorities, particularly African Americans. It is essential to address this difference in diagnosis to improve outcomes among racial minorities.

## Introduction

Pressure ulcer injuries (PIs), commonly known as bedsores or decubitus ulcers, are injuries to the skin and soft tissues due to ischemic changes caused by prolonged pressure on bony areas [1]. This condition is associated with increased pain, elevated risks of infection, inflated healthcare expenses, diminished quality of life for patients, and potentially even death [2]. Pressure ulcers are prevalent among hospitalized patients and significantly contribute to increased illness, mortality rates, and healthcare expenses. Despite being preventable, pressure injuries continue to be a significant cause of morbidity and financial burden in the United States [2]. Treatment options can be complicated, and there is a high likelihood of recurrence, especially for full-thickness wounds [3].

Social determinants of health have recently become a focal point of research, aiming to eliminate healthcare discrepancies between those in lower socioeconomic regions [4]. Studies have found that race and skin tone play crucial roles in the development and progression of pressure injuries. Some studies have shown that race has no predictive value about worse outcomes of patients with pressure injuries [5]. Then, some studies suggest that African American hospitalized patients are more likely to have higher-grade pressure injuries and recurrent pressure injuries compared to Whites [5,6]. However, these studies were limited by a lack of linking race with other social determinants of health. More literature must evaluate the prevalence and impact of pressure ulcer injuries.

This retrospective cohort study seeks to determine the prevalence of pressure ulcer injuries among various social groups and their effect on minorities among hospitalized patients in the US healthcare system. This article was previously presented as a meeting abstract at the 17th annual Medical Society State of New York (MSSNY) Resident/Fellow and Medical Student Poster symposium on April 12, 2024.

## Materials and methods

This retrospective cohort study comprises data from 2011 to 2020 from the National Inpatient Sample (NIS), which contains admission data of patients in the United States diagnosed with PI. An institutional review board (IRB) was not required for this study as the NIS includes patient information that has been de-identified and made publicly available. The data contain one primary diagnosis and up to 39 secondary diagnoses using the International Classification of Diseases, Tenth Revision (ICD-10) and 29 secondary diagnoses with the International Classification of Diseases, Ninth Revision, Clinical Modification (ICD-9-CM) codes [7]. Groups were defined based on the history of pressure injuries in their admission diagnosis, demographics, and comorbidities. Demographics, which were pulled from medical record data by the Healthcare Cost and Utilization Project (HCUP) when organizing the NIS data, were age, gender, race (White, African American, Latinx, Native American, Asian, and others), insurance status (private, Medicare, and Medicaid), and Charlson Comorbidity Index (CCI). CCI is a tool used to predict the 10-year survival in patients with multiple comorbidities; the comorbidities consist of age, myocardial infarction, congestive heart failure (CHF), peripheral vascular disease, cerebrovascular accident (CVA) or transient ischemic attack (TIA), dementia, chronic obstructive pulmonary disease (COPD), connective tissue disease, liver disease, diabetes mellitus, hemiplegia, solid tumor, leukemia, lymphoma, and AIDS [6]. The control group consisted of patients without pressure injuries, randomly selected by a random number generator once matched by comorbidities for each variable. A random selection was required due to the volume of patients who did not have pressure injuries and were admitted to hospitals in the United States, overloaded by the STATA software. For the confidence interval (CI) calculation, the comparison for sex was males, age 18-39 years, and for race Whites; insurance was private insurance; and CCI was a score of 0. The other treatment groups were then compared to see how these factors of sex, race, and insurance status changed a patient's outcome. The adverse outcomes measured were all-cause in-hospital mortality, sepsis, acute myocardial infarction (AMI), and acute kidney injury (AKI). 

The Pearson chi-square and Student's t-test assessed categorical and continuous variables. The linear-by-linear association test analyzed the trends in the frequency of pressure injuries in hospitalized patients. After adjusting for baseline characteristics and comorbidities to account for confounding variables, a two-step hierarchical multivariate regression model was used, emphasizing variables with p < 0.05. These variables included age, gender, race, Charlson Comorbidity Index (CCI), and insurance payer status. STATA version 17 by StataCorp LLC (College Station, TX) was utilized for all statistical analyses.

## Results

A weighted total of 5,993,667 PI admissions were included in this study and 5,993,667 non-PI admissions. PI patients were more likely than controls to be male (50.17% male versus 49.83% female; odds ratio {OR} = 0.88; 95% CI: 0.88, 0.89; males acted as the controls; p < 0.05). PI patients were also older than non-PI patients: age of 18-39 (5.55% versus 35.66%, p < 0.05), age of 40-64 (25.59% versus 28.27%, p < 0.05), age of 65-74 (21.98% versus 15.28%, p < 0.05), and age of 75+ (46.88% versus 20.80%, p < 0.05). Of the sample of PI patients, in ascending order of frequency, they were more frequently White (67.12% versus 65.45%, p < 0.05) than African American (19.65% versus 15.07%, p < 0.05), less frequently Latinx (8.06% versus 12.41%, p < 0.05), less likely to be Asians (2.01% versus 2.96%, p < 0.05), and less likely to be Native American (0.62% versus 0.62%, p > 0.05) (Table 1). 

**Table 1:** Demographic Data of Pressure Injury Patients From 2008 to 2020 P < 0.05. Native American control (0.62%) had a P of >0.05. Displayed values are the percent of groups. Groups: sex, age, race, insurance, and CCI CCI: Charlson Comorbidity Index

Weighted N in Sample		Pressure Ulcer Patients	Controls
		5,993,667	5,993,667
Sex	Male	3,007,023 (50.17%)	2,570,084 (42.88%)
	Female	2,986,644 (49.83%)	3,423,582 (57.12%)
Age	18-39	329,651 (5.50%)	2,137,341 (35.66%)
	40-64	1,533,669 (25.59%)	1,694,409 (28.27%)
	65-74	1,317,408 (21.98%)	915,832 (15.28%)
	75+	2,899,831 (46.88%)	1,246,682 (20.8%)
Race	White	4,022,949 (67.12%)	3,922,855 (65.45%)
	African American	1,177,755 (19.65%)	903,245 (15.07%)
	Latinx	483,089 (8.06%)	743,814 (12.41%)
	Asian	120,472 (2.01%)	177,412 (2.96%)
	Native American	37,160 (0.62%)	37,160 (0.62%)
	Others	152,838 (2.55%)	209,778 (3.5%)
Insurance	Private	596,969 (9.96%)	1,798,100 (30.06%)
	Medicare/Medicaid	5,214,490 (87.03%)	3,716,073 (62.09%)
CCI	0	59,936 (1.8%)	2,012,074 (33.57%)
	1-2	550,218 (9.18%)	1,089,648 (18.18%)
	3+	5,334,363 (89.02%)	2,876,960 (48.25%)

PI patients were more likely to be on Medicare or Medicaid (87.03% versus 62.09%; OR = 1.94; 95% CI: 1.92, 1.97; p < 0.05). PI patients were also far more likely to have a high Charlson Comorbidity Index of 3+ (89.02% versus 48.25%; OR = 10.44; 95% CI: 10.18, 10.71; p < 0.05) and far less likely to have a low CCI of 0 (1.80% versus 33.57%) or 1-2 (9.18% versus 18.18%; OR = 3.93; 95% CI: 3.84, 4.02; p < 0.05). Patients with higher CCI scores had higher odds of developing PIs (Table 1).

Males were more likely than females to develop pressure ulcers (adjusted OR = 0.88; 95% CI: 0.88, 0.89; p < 0.05). Compared to the patient in the age group 18-39, patients in older age groups were at higher risk of PI: age of 40-64 (OR = 2.09; 95% CI: 2.04, 2.13; p < 0.05), age of 65-74 (OR = 2.23; 95% CI: 2.18, 2.28; p < 0.05), and age of 75+ (OR = 3.46; 95% CI: 3.38, 3.54; p < 0.05). Compared to Whites, African Americans (OR = 1.49; 95% CI: 1.47, 1.51; p < 0.05) and Native Americans (OR = 1.26; 95% CI: 1.20, 1.32; p < 0.05) were at higher risk of PIs. However, Asians were at lower risk (OR = 0.94; 95% CI: 0.92, 0.97; p < 0.05). Latinx were at equal risk compared to Whites (OR = 0.99; 95% CI: 0.97, 1.01; p < 0.05) (Table 2).

**Table 2:** Demographic Pressure Ulcer Patients From 2008 to 2020: Odds Ratio and 95% Confidence Interval P < 0.05. P > 0.05 for Latinx odds ratio and 95% confidence interval. The odds ratio adjusted for confounding variables CCI: Charlson Comorbidity Index

Demographics	Categories	Overall (2011-2020)
Pressure ulcer patients		5,993,667
Sex	Male	1
	Female	0.88 (0.88-0.89)
Age	18-39	1
	40-64	2.09 (2.04-2.13)
	65-74	2.23 (2.18-2.28)
	75+	3.46 (3.38-3.54)
Race	White	1
	African American	1.49 (1.47-1.51)
	Latinx	0.99 (0.97-1.01)
	Asian	0.94 (0.92-0.97)
	Native American	1.26 (1.20-1.32)
	Others	1.08 (1.04-1.11)
Insurance	Private	1
	Medicare/Medicaid	1.94 (1.92-1.97)
CCI	0	1
	1-2	3.93 (3.84-4.02)
	3+	10.44 (10.18-10.71)

Compared to patients with private insurance, those with Medicare or Medicaid were at higher risk (OR = 1.94; 95% CI: 1.92, 1.97; p < 0.05). Patients with higher CCIs were also at higher risk than those with a CCI of 0: CCI of 1-2 (OR = 3.93; 95% CI: 3.84, 4.02; p < 0.05) and CCI of 3+ (OR = 10.44; 95% CI: 10.18, 10.71; p < 0.05) (Table 2).

Among PI patients, White patients had a lower risk of death compared to African Americans (OR = 1.09; 95% CI: 1.07, 1.11; p < 0.05), Latinx (OR = 1.16; 95% CI: 1.12, 1.19; p < 0.05), Asians (OR = 1.56; 95% CI: 1.50, 1.63; p < 0.05), and Native Americans (OR = 1.13; 95% CI: 1.03, 1.24; p < 0.05). African Americans had lower rates of AKI compared to Whites (OR = 0.88; 95% CI: 0.86, 0.91; p < 0.05). However, Whites had odds of AKI compared to Latinx (OR = 1.05; 95% CI: 1.01, 1.1; p < 0.05) and Asians (OR = 1.25; 95% CI: 1.18, 1.33; p < 0.05). Native Americans had an equal rate to Whites (OR = 0.94; 95% CI: 0.82, 1.07; p < 0.05). Compared to Whites, rates of sepsis were higher for African Americans (OR = 1.40; 95% CI: 1.38, 1.42; p < 0.05), Latinx (OR = 1.35; 95% CI: 1.32, 1.38; p < 0.05), Asians (OR = 1.79; 95% CI: 1.73, 1.85; p < 0.05), and Native Americans (OR = 1.14; 95% CI: 1.08, 1.2; p < 0.05) (Table 3).

**Table 3:** Pressure Injury Patient Outcomes From 2008 To 2020: Odds Ratio and 95% Confidence Interval P < 0.05. Odds ratio adjusted for confounding variables AMI, acute myocardial infarction; AKI, acute kidney injury; PI, pressure ulcer injury

Categories		Death	Sepsis	AMI	AKI
Number of PI patients		5,993,667	5,993,667	5,993,667	5,993,667
Sex	Male	1	1	1	1
	Female	0.91	0.88	0.87	0.87
Race	White	1	1	1	1
	African American	1.09	1.4	0.88	0.88
	Latinx	1.16	1.35	1.05	1.05
	Asian	1.56	1.79	1.25	1.25
	Native American	1.13	1.14	0.94	0.94
	Others	1.36	1.36	1.1	1.1
Insurance	Private	1	1	1	1
	Medicare/Medicaid	0.68	1.1	1.03	1.03

## Discussion

According to the National Pressure Injury Advisory Panel (NPIAP), a pressure ulcer is a localized damage to the skin and underlying soft tissue, usually over a bony prominence or related to medical or other devices due to intense or prolonged pressure or pressure combined with shear stress [8]. The most common locations for pressure injuries are the sacral and hip regions, with approximately 25% of all pressure injuries occurring on the lower extremities [9]. Our database comprises nearly six million hospitalized patients diagnosed with pressure injuries matched to controls without said condition.

In otherwise healthy individuals, prolonged pressure in a single area causes a feedback mechanism to cause a change in body position, preventing injuries. Individuals with abnormal sensation, mobility, and mental status may not possess this feedback mechanism, causing prolonged pressure and tissue injury [10]. Downward forces causing local ischemia from body weight onto bony prominences into surfaces such as wheelchairs, mattresses, or medical devices are the main culprits of pressure injuries [11]. A significant mobilization issue associated with pressure injuries is para- or quadriplegia due to a barrier causing a lack of mobility. Our results found that of those with pressure injuries, 12% were para- or quadriplegic compared to 0.3% of those without (p < 0.0001).

Another biochemical source of pressure injury is reperfusion injury after the blood supply has been restored due to free radical composition and inflammation [12]. Lastly, shearing forces and friction, such as patients placed at an incline and moisture from perspiration or incontinence, are additional well-understood mechanisms that can lead to the pathogenesis of pressure injuries [13,14].

In the hospital setting, pressure injuries are among the most common conditions acquired during acute stays or long-term care. In the United States, 1-3 million people will be affected annually by pressure injuries, and the rate among hospitalized patients ranges from 5% to 15% [15]. The national pressure ulcer prevalence survey of 2005 further broke down pressure injuries by specific location. It portrayed that ICUs had a higher rate of PIs at 25% compared to other hospitalized patients. Long-term acute care spaces also had a higher rate of PIs at 27% compared to general hospital patients [16].

All populations, ranging from children to adults, are at risk for pressure injury. Still, the elderly have the highest prevalence at 29% due to their many risk factors, with immobility caused by hospitalization being the most critical [17]. Our results found congruence to the overall prevalence rate, finding that those aged 65-74 had a rate of 22% and those aged 75+ had a rate of 47%. Interestingly, we found that 6% of patients aged 18-39 have pressure injuries, further showing how these injuries do not discriminate based on age. Other risk factors contributing to PIs include impaired sensation, malnutrition, and aging [10].

Despite an abundance of literature describing the pathogenesis, epidemiology, risks, and prevention strategies of pressure injuries, many of these articles fail to document and describe the discrepancies associated with race, age, sex, and insurance type. The presentation of pressure injuries and skin tone has been acknowledged in that one's skin tone will affect the classification of stage 1 pressure injuries [18]. According to the NPIAP, stage 1 is defined as non-blanchable erythema of intact skin. In those with darker skin tones compared to Caucasians, the blanching response is not as well-defined [18]. Also, dermatologic pathologies are not represented throughout various skin tones, leading to the misidentification of early-stage injuries and higher frequency of those in later stages [19].

As explained, we found that a significantly higher percentage of White patients have documented pressure ulcer injury compared to African Americans and even higher when compared to Latinx (67% versus 20% versus 8%, respectively). Native Americans had PI rates similar to White patients, possibly due to lighter skin tones (62% versus 67%). African Americans and Native Americans were significantly more likely to be at risk for the development of PIs compared to Whites (OR: 1.49 and 1.26). Still, interestingly, Asian populations had the lowest risk (OR: 0.94). These results align with the posed hypothesis that blanchable erythema in lighter skin tones can lead to the earlier and increased diagnosis of PI. Further investigation of whether these are high- or low-stage ulcers is necessary to substantiate our findings.

Males were more likely to develop PI than females (OR: 0.88), and the male sex seems to be a risk factor, which seems to be a consensus among the research. Still, some studies reference females as having higher rates of PI, and others find no significant difference [20]. A proposed theory as to why females may be at lower risk is due to their lower waist-to-hip ratio, larger hip circumference, and thicker gluteal subcutaneous fat, which may provide protective weight distribution to preserve blood flow in the sacral region [21].

Infection, specifically sepsis, is one of the most feared complications of PI. Annually, over 970,000 cases of sepsis are admitted, and the rates are continuing to rise year over year [22]. A prospective study among patients in a long-term care facility found that PIs were the second highest cause of bacteremia behind urinary tract infections [23]. PIs serve as a nidus for bacterial organisms such as methicillin-resistant *Staphylococcus aureus*, which pose a significant risk to patients especially when the injury is at a higher stage [24]. Impaired wound healing may be the only sign that infection is present, and thus, PIs must be cultured and treated with low clinical suspicion [25].

Our results found that sepsis rates were higher in African Americans, Latinx, Asians, and Native Americans compared to White patients (OR: 1.4, 1.35, 1.79, and 1.14, respectively) in patients with PIs. Previous research has shown that African American patients are more likely to develop higher-grade PIs than White patients, possibly explaining the higher rate of sepsis [5]. Individuals with Medicaid or Medicare were at a higher risk for PI compared to those with private insurance, and according to an article in JAMA, 56.4% of Medicaid patients were racial and ethnic minorities [26]. It is well-studied that patients with lower socioeconomic status (SES) have worse health outcomes, and that may be a reason for the racial disparity between PI and sepsis rates [27]. An article analyzing the relationship between race and socioeconomic conditions in the development of PIs after a spinal cord injury found that lower-income patients, who are more commonly Medicare and Medicaid patients, have higher rates of PIs [6]. It was hypothesized that they had less access to higher-quality care and less understanding of pressure injury prevention, leading to worse outcomes.

Moreover, we found a significant increase in PI among those with a higher Charlson Comorbidity Index, a validated score that classifies comorbid risks that predict mortality [28]. Those with a CCI of 3+ had the highest risk of PI than those with a score of 1-2, who were also higher than those with a score of 0. Some conditions measured in the CCI score related to PI pathogenesis are hemiplegia, diabetes mellitus, connective tissue disease, dementia, and peripheral vascular disease [29]. Therefore, it is reasonable to correlate higher scores with the likelihood of developing PI. This ties back to socioeconomic factors, where those with lower SES are more likely to have more comorbidities, leading to higher CCI scores and, thus, the increased risk of PI [9].

Our data depict significant disparities among various racial groups and socioeconomic factors. It is imperative that healthcare professionals become aware of these disparities and receive further training in spotting pressure injuries in darker skin tones in hopes of intervening at earlier stages. A minor improvement in PI incidence would relieve a significant burden on the US healthcare system, improving care for these populations.

This study has limitations that need to be addressed. The NIS dataset cannot trace patient encounters or admission diagnoses longitudinally. Also, the data are de-identified, so the frequency of admissions or the number of admissions of the same patient cannot be identified. Hence, problems can be over-calculated due to readmissions of the same patient. NIS data do not contain medication or medication history or laboratory values. NIS can only differentiate the main reason for admission, in this case pressure injuries using ICD-9 and ICD-10 codes. The ICD codes depend on healthcare workers' style of billing and coding, creating unknown confounders. Also, a transition was made in 2015 from ICD-9 to ICD-10 coding, which made another set of unknown confounders. The variability is also a potential confounder as variability exists in documentation. Finally, the NIS does not detail important clinical predictors, such as the severity of pressure injuries, the duration of comorbidities, and baseline function. The NIS does not have information on specific management. Since this is a retrospective cohort study, it has an inherent low external validity.

## Conclusions

The results of our study show that pressure injuries are more prevalent in patients with more debilitating comorbidities and older age, which is to be expected. However, this research highlights an ongoing racial discrepancy in pressure ulcer prevalence in patients of color, showing that racial minorities, particularly African Americans, are more likely to get pressure ulcers. This discrepancy might be partially explained by how low-grade pressure ulcers are defined, specifically by the enduring nature of non-blanchable erythema, which could result in an underdiagnosis in people with darker skin tones. This causes lower-stage injuries to go unnoticed, resulting in their progression. It is essential to address this difference in diagnosis to design specific treatments, the education of healthcare workers, and diagnostic criteria to improve outcomes for those who are at risk of pressure ulcers, particularly among racial minorities.
